# Eicosanoid Signaling in Insect Immunology: New Genes and Unresolved Issues

**DOI:** 10.3390/genes12020211

**Published:** 2021-02-01

**Authors:** Yonggyun Kim, David Stanley

**Affiliations:** 1 Department of Plant Medicals, College of Life Sciences, Andong National University, Andong 36729, Korea; 2 Biological Control of Insects Research Laboratory, USDA/Agricultural Research Service, 1503 South Providence Road, Columbia, MO 65203, USA; david.stanley@usda.gov

**Keywords:** prostaglandin, eicosanoid, immunity, insect

## Abstract

This paper is focused on eicosanoid signaling in insect immunology. We begin with eicosanoid biosynthesis through the actions of phospholipase A_2_, responsible for hydrolyzing the C18 polyunsaturated fatty acid, linoleic acid (18:2n-6), from cellular phospholipids, which is subsequently converted into arachidonic acid (AA; 20:4n-6) via elongases and desaturases. The synthesized AA is then oxygenated into one of three groups of eicosanoids, prostaglandins (PGs), epoxyeicosatrienoic acids (EETs) and lipoxygenase products. We mark the distinction between mammalian cyclooxygenases and insect peroxynectins, both of which convert AA into PGs. One PG, PGI_2_ (also called prostacyclin), is newly discovered in insects, as a negative regulator of immune reactions and a positive signal in juvenile development. Two new elements of insect PG biology are a PG dehydrogenase and a PG reductase, both of which enact necessary PG catabolism. EETs, which are produced from AA via cytochrome P450s, also act in immune signaling, acting as pro-inflammatory signals. Eicosanoids signal a wide range of cellular immune reactions to infections, invasions and wounding, including nodulation, cell spreading, hemocyte migration and releasing prophenoloxidase from oenocytoids, a class of lepidopteran hemocytes. We briefly review the relatively scant knowledge on insect PG receptors and note PGs also act in gut immunity and in humoral immunity. Detailed new information on PG actions in mosquito immunity against the malarial agent, *Plasmodium berghei*, has recently emerged and we treat this exciting new work. The new findings on eicosanoid actions in insect immunity have emerged from a very broad range of research at the genetic, cellular and organismal levels, all taking place at the international level.

## 1. Introduction

Immune reactions to invasions, infections and wounds are critical fitness traits in organisms, generally, and as the papers in this Special Issue illustrate, insects as well. While serious questions about the evolutionary ecology of microbial CRISPR-Cas immune systems remain open [1], the presence of these systems in bacteria and archaea and the differential expression of immune receptors in sponges (Order Porifera), the earliest extant metazoans [2], indicate immune mechanisms evolved very early in life. Immune reactions begin by recognition of pathogen-associated molecular patterns which launch biochemical signaling systems that activate insect cellular and humoral immune reactions. Insects express many immune-related signaling systems, such as Toll receptors, Immune deficiency (IMD), Janus Kinase and Signal Transducer and Activator of Transcription (JAK/STAT), biogenic amines and cytokines [3]. This paper lies in the context of a Special Issue devoted to insect immunology, which obviates a broad description of insect immunity.

Our work is focused on actions of prostaglandins (PGs) and other eicosanoids on insect immune signaling. Corey et al. [4] coined the term eicosanoid (from the Greek, εικοσι, the number 20) to describe all oxygenated metabolites of arachidonic acid (AA; 20:4n-6) and two other C20 polyunsaturated fatty acids (20:3n-6 and 20:5n-3). The three major groups of eicosanoids include PGs, epoxyeicosatrienoic acids (EETs) and various lipoxygenase products [5]. Members of all three groups are present and operate in insects. We reviewed eicosanoid biological actions in insects [6,7] and, more specifically, in insect immunity [8]. Eicosanoid chemical structures and biosynthetic pathways have been detailed in Stanley [9] and Stanley and Kim [6]. Interest in eicosanoid actions in invertebrates appears to be growing as new knowledge on eicosanoid signaling in insect immunity has emerged since drafting our last review. The purpose of this paper is to provide an updated understanding of eicosanoid actions in insect immunity. We develop a brief introduction to eicosanoid biosynthesis based on the biomedical background for perspective, and then treat what may be insect-specific PG biosynthesis. We shift focus onto specific eicosanoid actions in insect immunity. These include clearing bacterial infections from hemolymph circulation, nodulation, hemocyte spreading and release of prophenoloxidase (PPO) from specific lepidopteran hemocytes. We treat recent reports providing new information on PG actions in mosquito immunity.

## 2. Eicosanoid Biosynthesis

### 2.1. Phospholipases A_2_ (PLA_2_)

AA and other polyunsaturated fatty acids (PUFAs) are preferentially associated with phospholipids (PLs) that make up biological membranes, and they occur in much lower proportions in neutral, energy-storage lipids such as triacylglycerols. Various PLA_2_ are responsible for hydrolyzing PUFAs from PLs. On the biomedical background, there are five major PLA_2_ types, secretory (sPLA_2_), calcium-dependent (cPLA_2_), calcium-independent (iPLA_2_), lipoprotein-associated (LpPLA_2_) and adipose (AdPLA_2_) [10]. PLA_2_s exert a wide range of biological actions, such as dietary PL and neutral lipid digestion, remodeling cellular and subcellular membranes, signal transduction, host defense and, in mammals, pathophysiology, such as arthritis. cPLA_2_ and iPLA_2_ hydrolyze PUFAs from intracellular PLs, some of which are converted into eicosanoids. Park and Kim [11] revealed the broad biological significance of PLA_2_ actions in insect immunity with their discovery that eicosanoid treatments rescued beet armyworms, *Spodoptera exigua*, from lethal infections of the bacterium *Xenorhabdus nematophila*. They discovered that the bacterium somehow inhibited necessary PLA_2_ activity, required to release PUFAs from PLs for eicosanoid biosynthesis [12]. Park et al. [13] reported that *X. nematophila* inhibits PLA_2_s from insect, prokaryote and vertebrate sources. Indeed, a mutant generated by deleting a sPLA_2_ gene from the germline using CRISPR/Cas9 exhibits immunosuppression along with adverse effects on immature development and adult reproduction in a lepidopteran insect, *Maruca vitrata* [14]. We surmise PLA_2_s are of such biological power that infecting organisms evolve inhibitors of these enzymes, necessary for their survival.

We recently treated insect PLA_2_s in detail [7]. While there has been considerable progress on insect PLA_2_s, much more new knowledge is necessary on the biology, physiology, biochemistry and molecular biology of insect PLA_2_s.

### 2.2. Biosynthesis of AA

Mammals maintain substantial proportions of AA in PLs, from which it can be hydrolyzed for eicosanoid biosynthesis. Insects tend to maintain very low proportions of AA (often no more than trace amounts) and high proportions of linoleic acid (LA; 18:2n-6) in cellular PLs, which may help reduce oxidative damage to cellular PLs [15]. Elongation/desaturation pathways that convert LA into AA were documented in the 1980s [16], however the research was conducted in a biochemical, rather than a physiological context. Hasan et al. [17] reported three major advances in understanding PUFA metabolism. One, bacterial infections led to increased proportions of AA, up from undetectable in naïve *S. exigua* larvae to less than 1% following infections. The AA was produced via elongation/desaturation pathways, now documented at the molecular level in *S. exigua*. The increased AA was converted into eicosanoids, which signal cellular and humoral immune responses to infection. Two, unregulated AA biosynthesis, modelled by injecting larvae with free AA, was quite harmful to larvae, recorded as smaller pupal sizes and decreased egg production in adults. Three, the work described the *S. exigua* genes encoding the elongases and desaturases.

### 2.3. Mammalian Cyclooxygenases and Insect Peroxynectins (Pxts)

On the biomedical background, the 2-series PGs are biosynthesized from AA by a cyclooxygenase (COX), a protein with two catalytic sites. PG is the general abbreviation for prostaglandin. Specific PGs are denoted by a third letter, such as PGA_2_. The first converts AA into the endoperoxide, PGG_2_, and then a hydroperoxidase converts PGG_2_ into PGH_2_. Cell-specific enzymes convert PGH_2_ into any of several products, PGA_2_, PGB_2_, PGD_2_, PGE_2_, PGF_2α_, thromboxane B_2_ and PGI_2_ (also called prostacyclin). After genome databases became available, considerable efforts to identify one or more genes encoding COXs in insect genomes led to a conundrum. While PG actions including releasing cricket egg-laying behavior, modulating primary urine formation in isolated Malpighian tubules, signaling several aspects of insect immunity and post-translational protein phosphorylation [6,7,18], genes encoding insect COXs proved very elusive. For example, Varvas et al. [19] characterized two genes encoding COXs in two crustacean species, but not from insects. Tootle and Spradling [20] resolved the issue in their report that PGs mediate oogenesis in *Drosophila melanogaster.* The lack of genes encoding insect COXs is understood because a COX-like heme peroxidase, peroxynectin (Pxt), is responsible for PG biosynthesis. Park et al. [21] extended the idea of PG biosynthesis via Pxt in their report that two Pxts, SePOX-F and -H mediate, two aspects of cellular immunity in *S. exigua*, hemocyte spreading and nodule formation. The Pxts convert AA into PGH_2_, which is converted into other PGs by cell-specific enzymes [7]. Two Pxts, HPX7 and HPX8, are identified in a mosquito, *Anopheles gambiae*, in which they are likely to be associated with PG biosynthesis to mediate gut immune response against a malarial parasite infection [22].

Scarpati et al. [23] reported on applying a more rigorous approach to identifying genes encoding enzymes that act in eicosanoid biosynthesis in *D. melanogaster*. They used iterative machine learning and structural modeling to reveal “a surprising degree of similarity” between mammalian and fly eicosanoid biosynthesizing enzymes, which they grouped into high scoring matches, midrange candidates and the most distant candidates. For example, the gene CG1742 encodes a microsomal glutathione-S-transferase-like protein. The cognate protein also shares 36% identity and 54% similarity to the sequence of human prostaglandin E synthase (PGES), which was recently identified in *S. exigua* [24]. We note that the genes identified by Scarpati et al. [23] have not yet been shown to encode functional proteins that operate in eicosanoid biosynthesis. As it stands, the question of whether the identified genes act in *D. melanogaster* eicosanoid biosynthesis remains an unresolved issue.

### 2.4. PG Biosynthesis

**Figure 1:**
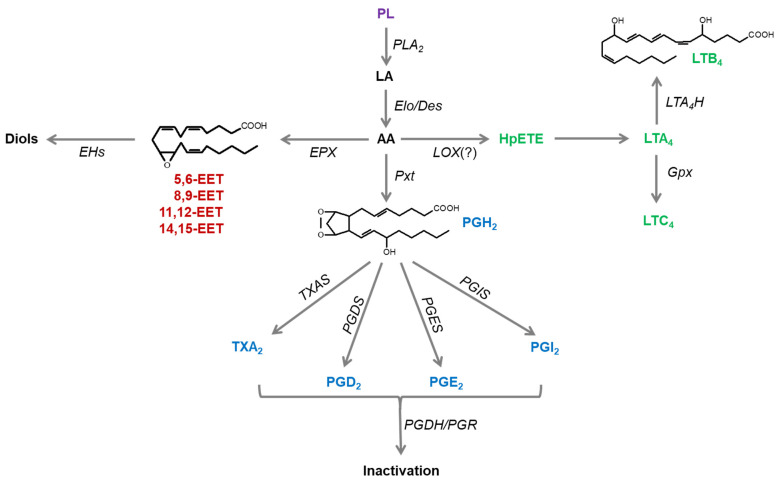
Eicosanoid biosynthesis and degradation in insects. Phospholipase A_2_ (PLA_2_) catalyzes hydrolysis of linoleic acid (LA) from membrane-associated phospholipids (PLs), which is elongated by long-chain fatty acid elongase (Elo) and desaturated by desaturase (Des) to arachidonic acid (AA). AA is then oxygenated by epoxidase (EPX) into epoxyeicosatrienoic acid (EET), lipoxygenase (LOX) into leukotriene (LT), or cyclooxygenase-like peroxynectin (Pxt) to prostaglandin (PG). EETs are degraded by soluble epoxide hydrolase (sEH). LTA_4_ is formed from 5-hydroxyperoxide eicosatetraenoic acid (HpETE) and changed into LTB_4_ by LTA4 hydrolase (LTA_4_H) or into LTC_4_ by glutathione peroxidase (Gpx). Various PGs are formed from PGH_2_ by cell-specific enzymes, thromboxane A_2_ (TXA_2_) synthase (TXAS), PGD_2_ synthase (PGDS), PGE_2_ synthase (PGES) and PGI_2_ synthase (PGIS). These PGs are degraded by PG dehydrogenase (PGDH) and PG reductase (PGR).

Based on similarity of insect eicosanoid biosynthesis to mammalian systems, two PG synthases were identified from *S. exigua* (Figure 1). SePGES and SePGDS are involved in biosynthesis of PGE_2_ and PGD_2_, respectively [24,25]. SePGES contains a consensus thioredoxin homology sequence (Cys-x-x-Cys) responsible for catalytic activity along with an N-terminal membrane-associated hydrophobic domain and a C-terminal cytosolic domain. It also shares sequence homology (36.5%) and shares almost overlapping three-dimensional structures with a membrane-bound human PGES2. SePGDS is also homologous (32.8%) to human PGDS. Based on its conserved active site residues, its N-terminal tyrosine (Y8) was predicted to mediate electron relay from glutathione to PGH_2_ substrate, which is distinct from the catalysis of SePGES. Both PG synthases are expressed in all developmental stages with high expression in late larval and adult stages. Individual RNA interference (RNAi) of *SePGES* or *SePGDS* expression suppressed cellular and humoral immune responses. The RNAi treatments also interfered with oocyte development in adults. In rescue experiments, the addition of PGE_2_ or PGD_2_ rescued the suppressed immune and reproductive responses. These bioinformatics and experimental results document the roles of these genes in *S. exigua* PG biosynthesis. We infer similar genes operate in insect PG biosynthesis, generally.

### 2.5. New Elements of Insect Oxylipins

Prostacyclin is also known as PGI_2_, the term we use in this review. This eicosanoid was discovered in 1976 and named PGX [26]. Among other roles, PGI_2_ is an active cardioprotective eicosanoid, acting as an inhibitor of platelet aggregation and a vasodilator [27,28]. Ahmed et al. (2021) used chemically elegant mass spectra to find that fat body from their model insect, *S. exigua*, contained PGI_2_ at about 3.6 pg/g tissue in untreated controls, which increased to nearly 5 pg/g tissue at 4 h post-bacterial challenge. They recorded mRNAs encoding a *S. exigua* PGI_2_ synthase (*SePGIS*) from all life stages, with relatively low expression in juveniles and higher expression in adults. The enzyme is expressed in hemocytes and fat body, with only traces in the larval gut. The authors recorded similar *SePGIS* expression levels in abdomen, testes and ovary and in thorax, with about 2-fold higher expression in females. Bacterial challenge stimulated increased accumulations of mRNAs encoding the protein in hemocytes and fat body. Ahmed et al. [28] generated a dsRNA construct to silence gene expression and showed that the RNAi treatments virtually obliterated expression.

The authors investigated possible PGI_2_ physiological actions in *S. exigua*. They found that treating bacterial-challenged larvae with dsRNA specific to *SePGIS* (dsPGIS) did not reduce hemocyte spreading relative to controls. However, dexamethasone (DEX) treatments, which inhibit all eicosanoid biosynthesis, led to about 4-fold reductions in spreading behavior in challenged larvae. This inhibition was not reversed in larvae treated with DEX + PGI_2_, and this finding indicates PGs other than PGI_2_ act in spreading. Treating experimental larvae with a bacterial challenge + PGI_2_ led to an approximately 25% decrease in hemocyte spreading, from which the authors inferred that PGI_2_ may be a negative regulator of cell spreading. They repeated their physiological experiments using another cellular immune reaction, nodulation, as their endpoint. This work returned similar findings, suggesting, again, that PGI_2_ is a negative regulator of insect cellular immunity.

Turning to the influence of PGI_2_ on development, Ahmed et al. [28] show that, compared to controls, dsPGIS treatments led to retarded development, seen as increased developmental periods for larvae and pupae, decreased pupation in fourth and fifth instar larvae and reduced body weights in larvae and pupae. dsPGIS treatments led to serious reductions in ovarian development. In more detail, dsPGIS treatments effectively stopped ovarian development, recorded as no development beyond the previtellogenic stage. This effect was rescued to a significant level, although not completely, by PGI_2_ treatments. Overall, the authors documented the presence of a new eicosanoid, PGI_2_, in a lepidopteran and demonstrated biological actions in immunity and development.

Vatanparast et al. [29] also introduced a new class of oxylipins in the biology of their model, *S. exigua*. These are oxygenated LA derivatives, epoxyoctadecamonoenoic acids (EpOMEs). For background, Vatanparast et al. [29] provided the chemical structures and outlined EpOME biosynthesis, beginning with release of LA from cellular PLs by a PLA_2_, an oxygenation step by a cytochrome P450 monooxygenase (CYP) and hydroxylation by an epoxide hydrolase. They determined the substantial presence of the compounds, nearly 1000 pg/g 9,10-EpOME and >2000 pg/g 12,13-EpOME in fat body. Injecting, separately, both EpOMEs into larvae led to dose-related reductions in hemocyte spreading and to reduced nodulation reactions in bacterial-challenged larvae. The EpOME treatments also influenced humoral immunity, with substantial reductions of mRNA encoding 10 anti-microbial peptides (AMPs). We infer that EpOMEs act in resolution of cellular and humoral immune reactions. 

### 2.6. PG Catabolism

Although PGs are essential for mediating cellular and humoral immune responses, uncontrolled and prolonged immune responses exert adverse effects on survival. Two PG-degrading enzymes, *PG dehydrogenase* (*SePGDH*) and *PG reductase* (*SePGR*), act in *S. exigua*. [30]. *SePGDH* and *SePGR* expression levels are upregulated after immune challenge. Gene expression peaks occurred after peaks of PG biosynthesis genes such as PGE_2_ synthase or PGD_2_ synthase. The inducible expressions of *SePGDH* and *SePGR* were specific to PGE_2_ or PGD_2_, but not to LTB_4_. RNAi treatment against *SePGDH* or *SePGR* expression led to excessive melanization and killed the larvae even after a non-pathogenic bacterial infection. The uncontrolled melanization in the RNAi-treated larvae was understood in terms of the prolonged PO activity by a bacterial challenge or PGE_2_ injection. The authors inferred that SePGDH and SePGR are responsible for the necessary PG degradation at a late phase of immune responses in insects.

### 2.7. EET Biosynthesis

EETs are a group of eicosanoids containing epoxide formed by CYP-catabolized epoxygenase (EPX) activity. Unlike dioxygenases such as COX or LOX, EPX is a monooxygenase that acts by inserting one oxygen into one of the four double bonds of AA to produce four metabolites: 5,6-EET, 8,9-EET, 11,12-EET, and 14-15-EET [31]. In mosquitoes, AA is an essential nutrient, required for development. Replacement of AA with PGs cannot meet the mosquito dietary requirement [32,33]. Indeed, mosquitoes are able to oxidize AA to form EETs, probably by CYP monooxygenases in in vivo or in vitro systems [34,35]. Subsequent chemical analysis of larval and adult mosquito tissues showed that three different EETs are present at 0.07~0.35 pmol/g [35].

Different EETs are biologically active in mammalian tissues [36]. 5,6-EET mediates somatostatin release from the hypothalamus [37]. In contrast, 14,15-EET mediates release of glucagon from pancreatic islets [38]. Dihydroxyeicosatrienoic acid (DHET) formed from 11,12-EET by a soluble epoxide hydrolase (sEH) inhibits a renal sodium-potassium pump [39]. In immunity, EETs are thought to be anti-inflammatory by attenuating cytokine-induced nuclear factor-κB activation and leukocyte adhesion to vascular walls [40], whereas DHETs are considered inactive or pro-inflammatory by activating nuclear factor-κB [41]. EETs are likely to act as pro-inflammatory factors because their enhanced levels stimulate expression of AMP genes and prevent pathogen load in mosquito midgut [42]. Along with EET detection in larval mosquitoes, immunological function of EETs in adults suggests that EETs are functional in other insects. This remains another unresolved issue because genes supporting possible roles of EETs in insect physiology remain unknown.

All four EETs (5,6-EET, 8,9-EET, 11,12-EET, and 14,15-EET) have been identified in *S. exigua* in larval fat body at 247~1736 pg/g levels [43]. To explore their biosynthesis, genes encoding 140 CYPs were collected from *S. exigua* transcriptomes and compared with human EPXs. EETs are CYP metabolites synthesized from AA, in which the main AA-metabolizing CYPs are CYP1A, CYP2B, CYP2C, CYP2E, CYP2J, and CYP3A4 in human [34,44]. Four CYPs (*SeEPX1*-*SeEPX4*) sharing homologies with human EPXs were predicted and subsequent expression and functional analyses documented their association with immune responses. These candidates feature CYP-conserved domains, such as oxygen and iron binding domains, and clustered with human CYP2 family EPXs. They are predominantly expressed in immunity-associated tissues, fat body and hemocytes, and their expression levels were highly enhanced by bacterial challenge. RNAi treatments interfered with hemocyte-spreading behavior and nodule formation upon bacterial challenge, except RNAi treatment against *SeEPX2*. The RNAi-suppressed immune responses against three *SeEPX*s were rescued by the addition of 8,9-EET. However, the three other EETs gave their specific rescue effect depending on *SeEPX* types under RNAi. In humoral immune responses, all four RNAi treatments suppressed expression of antimicrobial peptide genes. This study reports the presence of all four EETs in larval fat body of *S. exigua* and suggests that four SeEPXs are associated with immune responses mediated by EETs.

## 3. Eicosanoid Actions in Insect Immunity

### 3.1. Clearing Bacteria from Hemolymph

Stanley-Samuelson et al. [45] tested the hypothesis that eicosanoids mediate insect immune reactions to bacterial infections. They used a classical pharmaceutical approach of treating 5th instar tobacco hornworms, *Manduca sexta*, with an inhibitor of eicosanoid biosynthesis (DEX), and then challenging the larvae with a bacterial infection. They used a red-pigmented strain of the Gram-negative bacterium, *Serratia marcescens*. After selected incubation periods, they withdrew hemolymph samples, streaked the samples on agar plates over-night, then counted numbers of red-pigmented colony-forming units (CFUs). While no pigmented CFUs were recovered from control larvae, substantial numbers of pigmented CFUs were recovered from DEX-treated larvae. A separate experiment revealed higher mortality, compared to controls, in the experimental larvae. The authors concluded that eicosanoids mediate clearance of infecting bacteria from hemolymph, without a speculating on a specific mechanism.

### 3.2. Nodulation

Miller et al. [46] used a similar experimental design to find that eicosanoids mediate cellular micro-aggregation and nodulation reactions to *S. marcescens* infection, also in *M. sexta* larvae. In the work, experimental larvae were separately treated with a range of pharmaceutical eicosanoid biosynthesis inhibitors and challenged with bacterial infection. At selected times post-infection, nodulation reactions were quantitatively assessed by counting discrete nodules. Subsequent work by several groups confirmed eicosanoids mediate nodulation reactions in a broad range of species from several orders (reviewed in Stanley [47]). We infer that nodulation reactions clear infecting bacteria from hemolymph circulation in insects, generally.

### 3.3. Cell Spreading

Nodulation reactions to infections involve cell spreading, the significance of which was demonstrated by identification of a plasmatocyte spreading peptide (PSP) from hemolymph of the lepidopteran, *Pseudoplusia includens* [48]. This was the first known insect cytokine. Miller [49] investigated the idea that eicosanoids act in plasmatocyte spreading. He injected DEX into tobacco hornworms, *M. sexta*, then withdrew hemolymph samples and allowed plasmatocytes to spread on glass slides. DEX-treated cells did not elongate to the extent recorded for hemocyte preparations from control insects. The DEX effect was expressed in a dose-related manner and reversed by injecting AA into DEX-treated insects. The author suggested plasmatocyte spreading was influenced by eicosanoids. Srikanth et al. [50] advanced this work with their finding that PSP acts via PGs in hemocyte preparations from *S. exigua*. The authors found that treating hemocyte preparations with PSP and, independently, with PGs, led to cell spreading and that treatments with pharmaceutical inhibitors of PG biosynthesis reversibly blocked the process. They also found that silencing the gene encoding proPSP with its specific dsRNA construct blocked hemocyte spreading, which could be reversed by the addition of PSP and, separately, AA treatments. They suggested a signaling model in which PSP acts through a cell surface PSP receptor to increase biosynthesis of PGs that mediate plasmatocyte spreading. This crosstalk between PSP and PGs is explained by a small G protein, Rac1, a member of the Rho family including Cdc42, Rho and Rac, known to mediate cytoskeletal rearrangements in vertebrates [51]. Hemocyte spreading behavior requires actin remodeling to form filopodial or pseudopodial cytoplasmic extensions. In *Drosophila*, PGs activate fascin for actin filament-bundling to rearrange cytoskeletons [52]. In *S. exigua*, Rac1 mediates F-actin growth in hemocytes and also activates PLA_2_ [53], which leads to PG biosynthesis. In contrast, RNAi of *Rac1* expression interfered with the hemocyte spreading behavior in response to PSP in *S. exigua* [53]. This clarifies the crosstalk between PSP and PGs with respect to Rac1 activation by PSP, which subsequently increases PG concentrations for actin remodeling for hemocyte spreading behavior.

**Figure 2:**
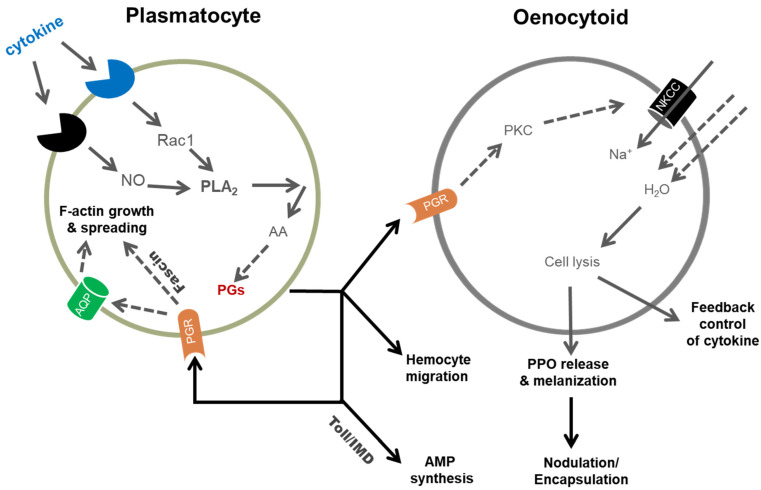
Prostaglandins (PGs) mediate cellular and humoral immune responses through specific receptors (PGRs). PGs are synthesized from arachidonic acid (AA), which is released from phospholipids by phospholipase A_2_ (PLA_2_) and is activated by immune challenge via cytokines or nitric oxide (NO). Oenocytoid cell lysis is mediated by PGR through a PKC pathway to activate a sodium-potassium-chloride cotransporter (NKCC), which generates an osmotic gradient that pulls water into the cell. Prophenoloxidae (PPO) is released from oenocytoid into hemolymph, where it is activated to phenoloxidase, which leads to melanin formation. In plasmatocyte, PGR activates cytoskeletal rearrangement via a small G protein Cdc42 and change cell volume via aquaporin (AQP) for hemocyte-spreading behavior. PGs also mediate antimicrobial peptide (AMP) synthesis via Toll/IMD immune signaling components.

The PG-Fascin-actin remodeling pathway does not fully explain the underlying molecular processes driving hemocyte spreading behavior. For example, phagocytosis requires extensive cell spreading and it is one of the cellular immune responses to defend against bacterial infection [54]. In cytoplasmic extension, a sequential process of actin cytoskeletal rearrangement, including elongation of F-actin, its branching and bundling, are required for formation of filopodia or lamellipodia [55]. Several actin-associating factors such as profilin, Arp2/3, Enabled and fascin play crucial roles in protruding hemocyte cell membrane in *Drosophila* [56,57]. Here is the question whether the small G proteins including Rac1 activate the actin-associating factors in the hemocyte spreading. Based on Fascin, a molecular component of PGE_2_ signaling pathway in *D. melanogaster* [52], protein–protein interactions were predicted using the computer modeling program, STRING (http://version10a.string-db.org), and showed that Fascin interacts with 20 proteins including another Rho family small G protein, Cdc42 [30]. In the binding model, Cdc42 interacts with several actin-associated factors including actin monomer, actin-related proteins (Arp2/3) and profilin. With an addition of cofilin due to its function in actin remodeling [58], fascin, Arp2, profilin and Cdc42 were predicted as actin-associated factors that could lead to hemocyte spreading behavior in response to PGE_2_ [30]. Under individual RNAi treatments, the hemocyte spreading behavior was significantly impaired, except for dsRNA treatment against *cofilin*, an actin-depolymerizing factor. Interestingly, the altered cytoskeleton patterns induced by the RNAi treatments were different. RNAi against *Arp2* markedly suppressed lamellipodial extension, while RNAi against *Profilin* or *Fascin* suppressed filopodial extension. Moreover, these RNAi treatments prevented the PGE_2_ modulation of hemocyte spreading behavior, suggesting that PGE_2_ mediates the cell spreading via Cdc42 to activate downstream actin polymerization/branching/bundling factors in a molecular process of actin cytoskeletal rearrangement (Figure 2).

In addition to actin cytoskeletal rearrangement, the hemocyte spreading behavior is accompanied by cell volume change at leading ends. A hypothesis of transmembrane water transport was raised to explain the change of the local cell volume [59]. A water-transporting pore (aquaporin: AQP) has been identified in *S. exigua* and its cellular locality in the cell membrane was confirmed. Hemocytes in RNAi-treated larvae did not change cell volume under hyper- or hypo-tonic osmotic stresses. The RNAi treatment also impaired cellular immune responses such as phagocytosis and nodule formation upon PGE_2_ treatment [59]. The authors inferred that PGs mediate hemocyte spreading behavior by activating AQP and actin-cytoskeletal rearrangement upon immune challenge.

### 3.4. Releasing Prophenoloxidase (PPO) from Oenocytoids

PPO is released from oenocytoids, a class of lepidopteran hemocytes, into circulating hemolymph. Recognition of pathogens or parasites activates serine proteases, enzymes in hemolymph that activate PPOs into POs by proteolytic cleavage. POs launch a train of serine protease activation reactions that lead to synthesis of melanin, an important component of insect immunity [60]. Melanin can be deposited on newly formed hemocyte nodules and on parasites after hemocyte encapsulation, where, as a chemically active compound, it helps kill pathogens. Melanin is also deposited on wound sites to protect from possible infections and invasions [61,62]. Melanization is an important component of insect host defense.

Shrestha and Kim [63] raised a key question on the release of PPO from hemocytes. They reported that PGs mediate release of PPO from oenocytoids in *S. exigua*. This work added an important new PG function in insect immunity. They followed up on their finding by investigating the mechanism of releasing PPO, which led to discovery of the first known insect PG receptor [64]. In this work, they reported the receptor, Se-hcPGGPCR1 amino acid and nucleotide sequences, showed it is expressed in all life stages and that accumulations of mRNAs encoding the receptor are vastly increased at 4 h post-infection. In situ hybridization showed that the receptor is expressed solely in oenocytoids, not in plasmatocytes nor granular cells. They treated experimental larvae with a dsRNA construct specific to the receptor, which led to reduced PO activity and to reduced oenocytoid cell lysis (OCL). They inferred that PGE_2_ mediates OCL via its specific receptor. Shrestha et al. [65] drilled deeper into the OCL mechanism. They found that PGE_2_ activates a sodium channel, a sodium-potassium-chloride cotransporter 1 (SeNKCC1), which is expressed in hemocytes. Treating experimental larvae with a dsRNA construct against SeNKCC1 led to reduced PGE_2_-stimulated OCL. In a direct test of the co-transporter action in an immune parameter, the authors recorded reduced nodule formation following bacterial infection. Hence, SeNKCC1 is necessary for OCL and for at least one cellular immune reaction to infection.

We note that compared to the biomedical background, there is scant information on insect eicosanoid receptors. Aside from the receptor just discussed, Kwon et al. [66] reported on a PGE_2_ receptor in *M. sexta*. The receptor is similar to human EP2 receptor and specifically expressed in oenocytoids. A similar PGE_2_ receptor was also identified in *S. exigua* and following its heterologous expression in Sf9 cells, it responded to PGE_2_ treatment by elevating cAMP via a trimeric G protein, Gαs [67]. A deletion mutant of the PGE_2_ receptor by CRISPR/Cas9 led to significant immunosuppression along with retarded larval growth and adult ovarian development.

Kwon et al. [68] posted a preprint on the Biorxiv preprint server, in which they report on a PGE_2_ receptor (AgPGE_2_R) that regulates mosquito, *A. gambiae*, oenocytoid immune cell function. The receptor is expressed in midgut, fat body, ovary, Malpighian tubules and hemocytes. In naïve mosquitoes, high genes’ expression was recorded in Malpighian tubules and hemocytes. At 24 h post-blood meal, highest expression occurred in Malpighian tubules, and expression was generally suppressed in *P. berghei*-infected mosquitoes. Western blot analysis showed bands at 70 kDa, representing a glycosylated AgPGE_2_R, in blood-fed but not *Plasmodium*-infected hemolymph. Immunofluorescence assays showed the receptor is expressed in oenocytoids, but not other hemocytes. PGE_2_ treatments led to increased expression of PPO3, 7 and 8. RNAi treatment with a specific dsRNA (dsPGE2R) to *AgPGE_2_R* construct led to reduced expression of these three PPOs. PGE_2_ treatments led to increased PO activity. PGE_2_ priming resulted in significant reductions in oocyst numbers compared to controls, while dsPGE2R treatments had the opposite influence. This work adds new information on anti-plasmodium immunity and contributes information on a third known insect PG receptor.

### 3.5. Hemocyte Migration

Hemocyte migration is an integral feature of cellular immune reactions. Hemocytes undergo directed migration toward sites of microbial infection and wounding. Merchant et al. [69] reported on the outcomes of experiments designed to test two ideas. First, insect hemocytes are able to detect and migrate toward a source of N-formyl-Met-Leu-Phe (fMLP). fMLP is a chemotactic peptide produced by the Gram-negative bacterium *Escherichia coli* and it is responsible for attracting neutrophils, which are produced in mammalian bone marrow [70]. Second, hemocyte migration is mediated by eicosanoids. In separate experiments, the authors treated *M. sexta* larvae with pharmaceutical inhibitors of eicosanoid biosynthesis, DEX and indomethacin (INDO), then prepared primary hemocyte cultures. Hemocyte migration was measured in Boyden blind-well chambers (illustrated in Merchant et al. [69]). In control experiments, about 42% of hemocytes migrated across small-pore membranes toward saline; compared to controls, about 64% migrated toward fMLP. Migration was reduced in a dose-dependent manner in hemocyte preparations from larvae treated with DEX or INDO. The failed migration was rescued in hemocytes prepared from larvae treated with DEX plus the eicosanoid precursor fatty acid, AA. Merchant et al. [69] inferred that hemocytes are able to detect and respond to fMLP and that insect hemocyte migration is mediated by eicosanoids.

### 3.6. PG Actions in Gut Immunity

In insect midgut, PGs play a crucial role in defending against microbial pathogens. Dual oxidase (Duox) is a main immune executor for gut immunity in insects by producing reactive oxygen species (ROS) [71]. In *S. exigua,* a Duox gene (*Se-Duox*) is expressed in the midgut of late larval instars and is upregulated by a bacterial challenge [72]. In this study, RNAi of *Se-Duox* expression significantly suppressed ROS amounts in the midgut lumen. Interestingly, treatments with a PG biosynthesis inhibitor significantly suppressed *Se-Duox* expression and addition of PGE_2_ or PGD_2_ rescued the inhibition. The signaling pathway from PGE_2_ to *Se-Duox* expression likely involves cAMP and its downstream components because specific inhibitors of cAMP signal components, adenylate cyclase (AC) and protein kinase A (PKA), significantly inhibited *Se-Duox* expression. Indeed, addition of a cAMP analog stimulated *Se-Duox* expression in the midgut. Furthermore, individual RNAi specific to a PGE_2_ receptor (a trimeric G protein subunit), AC, Protein Kinase A, or cAMP-responsive element-binding protein resulted in suppression of *Se-Duox* expression. These results suggest that PGs act in gut immunity by inducing *Duox* expression in insect gut to produce antimicrobial ROS. However, it remains unknown whether PGs act in the molecular signaling processes to activate *Duox*.

### 3.7. Eicosanoid Actions in Humoral Immunity

Here, we link the Toll/IMD pathways with eicosanoid signaling, detailed in Stanley and Kim [7]. The key point is that Toll and IMD activate PLA_2_, which leads to eicosanoid biosynthesis and actions. Two mechanisms operate in the PLA_2_ activation. One is upregulating expression of genes encoding PLA_2_ and the other involves translocation of the enzyme from the cytosol to the membrane-linked PLs. The key point of this brief section is that eicosanoids act in humoral, as well as cellular immunity.

The first report on the functional crosstalk between Toll/IMD immune signaling pathway and eicosanoids was the AMP gene expression in *Drosophila* [73]. Lipopolysaccharide (LPS) activates AMP gene expression under the IMD signaling pathway in this insect. However, treatments of specific PLA_2_ inhibitors suppressed the AMP expression under LPS exposure. An addition of PLA_2_-catalytic products rescued the AMP expression, though the single treatment of the products without LPS did not induce the AMP expression. Shrestha and Kim [74] further analyzed the crosstalk of the immune signal pathways using *Tribolium castaneum*, a well-known RNAi-responsive model insect. In this system, also, bacterial challenge significantly upregulated PLA_2_ activity in the larvae. However, the induction of the enzyme activity was not observed when the larvae were treated with dsRNAs specific to Toll or IMD genes. Furthermore, the RNAi treatments also suppressed the induction of *PLA_2_* expression upon the bacterial challenge. The functional links were further supported by the role of Toll/IMD signal in PLA_2_ intracellular translocation. Upon bacterial challenge, PLA_2_s were observed nearer to cell membrane of hemocytes in control larvae, but hemocytes collected from larvae treated with the dsRNAs specific to Toll or IMD genes did not show the translocation, at which the PLA_2_s appeared to be evenly spread in the cytoplasm. To explore the molecular action to link the Toll/Imd pathway to PLA_2_ activation upon immune challenge, Toll signal components, MyD88 and Pelle, were assessed in their functional interaction with PLA_2_ [75]. MyD88 possesses a Toll/Interleukin receptor domain to interact with Toll receptor and Tube/Pelle to activate expression of some genes encoding AMPs as a response to microbial infection [76,77]. In a functional trimeric (MyD88-Tube-Pelle) complex, Pelle kinase activity phosphorylates Cactus, an inhibitor ĸB (IĸB) factor, for its degradation to facilitate nuclear translocation of NF-ĸB transcription factor(s) such as Dorsal/Dif in *Drosophila* for production of specific AMPs [78]. Thus, MyD88 and Pelle play crucial roles in the Toll signal pathway. These two genes were identified in *S. exigua* and their RNAi treatments suppressed the upregulation of PLA_2_ enzyme activity and its gene expression under immune challenge [75]. Immunosuppression induced by RNAi of Toll signal molecules was significantly reversed by AA addition. These results document the crosstalk between Toll and eicosanoid signals in insect immunity.

### 3.8. Eicosanoid Actions in Mosquito Immunity

Barletta et al. [22] investigated the idea that PGE_2_ is a biochemical signal that attracts hemocytes to the basal surfaces of mosquito, *A. gambiae*, midgut cells. In brief, the authors determined PG concentrations in hemolymph from sugar-fed, blood-fed and *P. berghei* ookinete (a mosquito-borne developmental stage of the malaria pathogen) fed mosquitoes. They recorded a small, significant increase in hemolymph PG concentration at 24 h post-blood meal. Feeding on a *P. berghei*-infected mouse led to a substantial, approximately 6-fold, increase in hemolymph PGE_2_ concentrations. The *P. berghei* ookinetes disrupt the midgut peritrophic membranes, which facilitates direct contact between the microbiome bacteria and the lumen side of midgut epithelial cells. The authors recorded PGs in midguts, over 200 pg/mL in hemolymph without bacterial contact and about 500 pg/mL in midguts exposed to bacteria. They also show PGE_2_ immunostaining in midguts after, but not before, bacterial contact. The PGs expressed in midguts led to recruitment of hemocytes to the hemolymph side of midguts. They used time-lapse imaging to document hemocyte migration toward PGE_2_. They found that feeding mosquitos with bacterial-laden bovine serum albumin induces expression of two heme peroxidases, HPX7 and HPX8, responsible for PG biosynthesis. Silencing these genes prevents the increase in PG release after blood feeding. Overall, the authors demonstrated that ingesting *P. berghei*-infected blood led to increase PG biosynthesis, which attracted hemocytes to midgut surfaces and increased their patrolling activity.

Their work demonstrated the action of another eicosanoid in mosquito immunity [22]. Contact between the mosquito microbiome and midgut epithelial cells also leads to systemic release of a hemocyte differentiation factor (HDF) which has two components, the eicosanoid Lipoxin A_4_ (LPA_4_), bound to a lipocalin carrier. HDF increases proportions of circulating hemocytes, which are necessary for patrolling the midgut epithelia. LPA_4_ is derived from AA by sequential LOX actions. In one pathway, 5-lipoxygenase converts AA into 5-hydroxyperoxyeicosatetraenoic acid (5-HPETE), and then 5-lipoxygenase converts 5-HPETE into leukotriene A_4_ (LTA_4_), which is converted into the biologically active LTB_4_. The actions of these two eicosanoids leads to enduring systemic cellular reactions to *P. berghei* infection.

In a related study, Kwon and Smith [79] reported the outcomes of injecting inhibitors of eicosanoid biosynthesizing enzymes into females of the mosquito, *A. gambiae,* on survival of *P. berghei* oocysts. Experimental mosquitoes were treated by feeding on *P. berghei*-infected mice and control were fed on uninfected mice. They found that the PLA_2_ inhibitor, DEX, and the COX inhibitor, indomethacin, did not influence internal oocyst populations. Injecting the LOX inhibitor, esculetin, led to significant increases in oocyst numbers and treating mosquitoes with the epoxide hydrolase inhibitor, 12-[[(tricyclo [3.3.1.13,7]dec-1-ylamino)carbonyl]amino]-dodecanoic acid (AUDA), led to steeply reduced oocyst numbers. The AUDA findings bring up another group of oxylipins, the oxygenated metabolites of linoleic acid (LA; 18:2n-6). LA is converted into four oxylipins, two of which we mention here, 9,10-epoxyoctadecenoic acid (9,10-EpOME) and 12,13-EpOME. These two compounds occur in *S. exigua*, where they act in attenuating immune reactions in late infection [43]. Both EpOMEs are produced by cytochrome P450s and catabolized by soluble epoxide hydrolases (sEHs) that convert the epoxy rings into diols [80]. sEHs also act in catabolism of a group of eicosanoids, the EETs. Xu et al. [42] suggested that epoxy fatty acids are taken into midguts with blood meals, where they influence microbes. A mechanism of how the AUDA treatments influence oocyte survival remains unresolved.

Working with another mosquito species, *Aedes aegypti*, Barletta et al. [81] identified 40 immunity-conferring genes in *A. aegypti,* including several genes encoding AMPs and a Class C scavenger receptor. Expression of some of these genes, e.g., cecropin, was upregulated in the presence of ASA and others, e.g., transferrin, were downregulated. In mosquitoes infected with *Enterobacter cloacae*, Dengue virus or Sindbis virus, the numbers of microbes were increased in the presence of ASA. The authors found that inhibition of PG biosynthesis in midguts led to decreased expression of several AMPs. They identified six *A. aegypti* genes encoding PLA_2_s classified into sPLA_2_ and iPLA_2_ and showed that blood feeding led to increased expression of PLA_2_c. The authors concluded that PGs make up an important component of mosquito immune reactions to bacterial and viral infection. Their conclusion is rather subtle, indicating the PG are not responsible for activating immune responses, but act in modulating the amplitude of the response.

## 4. Prospectus

A hypothesis that eicosanoids mediate immune responses has been confirmed in 29 or so insect species from seven orders [7]. Current efforts identified unknown prostanoids and other eicosanoids in insect tissues. Their biosynthetic machineries have been unraveled. In addition, the identification of PGE_2_ receptors shed light on understanding fundamental signaling system in insect immunity. These biochemical and molecular processes mediated by eicosanoids have been applied to medically important mosquitoes to understand their interactions with parasites. Despite the scientific advances, a number of fundamental questions remain unanswered in insect eicosanoids. First, various eicosanoids classified into PGs, LTs and EETs mediate cellular immune responses such as nodule formation. Although PGI_2_ acts as a resolving mediator [28], most eicosanoids activate the immune responses. Furthermore, C18 oxylipins also mediate immune responses in insects, in which EpOMEs negatively mediate the immune responses [29]. Their metabolized products called dihydroxy-octadecamonoenoates (DiHOMEs) are also detected in mosquito [35]. These C18 oxylipins, in addition to eicosanoids, suggest that various PUFAs have their roles in insect immunity and opens a new research area in insect physiology on oxylipins. Second, so far, none of the LTs are identified in insects. However, LTB_4_ mediates cellular and humoral immune responses in insects [8]. Furthermore, Scarpati et al. [23] predicts LTA_4_ synthase in *D. melanogaster*. However, the known insect genomes do not encode mammalian lipoxygenase (LOX) genes. These suggest that insects may possess unique type of LOX genes. Third, eicosanoids act as a common downstream signal of other immune mediators, such as biogenic monoamines, nitric oxide and cytokines [53]. However, there is no study on the specific crosstalk between eicosanoid types and different immune mediators to perform specific functional associations in different tissues and developmental stages. It is reasoned why they use eicosanoids as downstream signals because the various chemical types of eicosanoids compared to the other immune mediators may mediate sophisticated immune processes. Last, the recent mosquito work advances understanding of insect immunology generally, while showing subtle mosquito-specific aspects of eicosanoid signaling. We look forward to up-coming advances in the area.
